# Investigation of Underlying Association Between Whole Brain Regions and Alzheimer’s Disease: A Research Based on an Artificial Intelligence Model

**DOI:** 10.3389/fnagi.2022.872530

**Published:** 2022-06-07

**Authors:** Shui Liu, Chen Jie, Weimin Zheng, Jingjing Cui, Zhiqun Wang

**Affiliations:** Department of Radiology, Aerospace Center Hospital, Beijing, China

**Keywords:** Alzheimer’s disease, magnetic resonance imaging, radiomics, machine learning, structural MRI (sMRI)

## Abstract

Alzheimer’s disease (AD) is the most common form of dementia, causing progressive cognitive decline. Radiomic features obtained from structural magnetic resonance imaging (sMRI) have shown a great potential in predicting this disease. However, radiomic features based on the whole brain segmented regions have not been explored yet. In our study, we collected sMRI data that include 80 patients with AD and 80 healthy controls (HCs). For each patient, the T1 weighted image (T1WI) images were segmented into 106 subregions, and radiomic features were extracted from each subregion. Then, we analyzed the radiomic features of specific brain subregions that were most related to AD. Based on the selective radiomic features from specific brain subregions, we built an integrated model using the best machine learning algorithms, and the diagnostic accuracy was evaluated. The subregions most relevant to AD included the hippocampus, the inferior parietal lobe, the precuneus, and the lateral occipital gyrus. These subregions exhibited several important radiomic features that include shape, gray level size zone matrix (GLSZM), and gray level dependence matrix (GLDM), among others. Based on the comparison among different algorithms, we constructed the best model using the Logistic regression (LR) algorithm, which reached an accuracy of 0.962. Conclusively, we constructed an excellent model based on radiomic features from several specific AD-related subregions, which could give a potential biomarker for predicting AD.

## Introduction

Alzheimer’s disease (AD) is the most common form of dementia, characterized by episodic memory decline. Its incidence is rising as the population ages and there are approximately 50 million people suffering from AD worldwide at present, which imposes a heavy burden on the society (Nichols et al., 2019; Breijyeh and Karaman, 2020). Due to the lack of sensitive diagnoses and effective treatments, it is of great theoretical significance and of potential clinical value to establish reliable radiologic biomarkers for early detection of AD by using new technologies, which can improve the prognosis of the disease.

Neuroimaging studies in AD have revealed the relationship between AD and structural atrophy in the temporal lobe, the entorhinal cortex, the hippocampus, and the limbic system, which reflects the different stages of the disease and predicts the progress of AD (Sørensen et al., 2016; Wolk et al., 2017; Li et al., 2018). Previous studies used the analysis based on manually labeled regions of interest (ROI) to explore the subtle structural atrophy leading to AD (Last et al., 2020). In recent years, machine learning provides an automated and objective classification framework that includes feature extraction, algorithm selection, predictive model building, model validation, and so on. In the AD classification field, machine learning has attracted increasing attention by using the multimodal quantify patterns of atrophy together with different algorithms in recent years (Tang et al., 2015). For the radiomic feature extraction, brain atrophy was most often quantified *via* volume, texture, and geometric shape measures from structural magnetic resonance imaging (sMRI), which has achieved promising results.

For instance, one study combined hippocampal volume information and machine learning, suggesting that the volumetric reduction in the hippocampus was an important indicator of early AD (Uysal and Ozturk, 2020). Texture analysis had been successfully employed to search for imaging biomarkers for AD (De Oliveira et al., 2011; Anandh et al., 2015; Chincarini et al., 2016). Several studies had confirmed hippocampal texture abnormalities in AD and early stages of AD, indicating that texture might predict early cognitive impairment (Sørensen et al., 2016). Recently, Feng et al. employed the radiomic features of hippocampal subregions using a support vector machine (SVM) model to distinguish AD from healthy control (HC) and showed good performance (Feng et al., 2018). Besides the hippocampus, one study analyzed the texture features of the corpus callosum and the thalamus, suggesting that these regions could be used for the early diagnosis of AD (De Oliveira et al., 2011). Another study used the random forest (RF) classifier to identify the subcortical regions and found that the radiomic features from the hippocampus and amygdala regions have the greatest discriminative ability, which could differentiate AD from HC with the best performance (Chaddad et al., 2018). For geometric shape measures, a recent study used the geometric shape of the corpus callosum and multilayer perceptron (MLP) classifier to differentiate AD from HC, in which the classifier showed a high accuracy value (Dadsena et al., 2019).

As listed above, the research of AD prediction based on structural radiologic features and machine learning has made promising progress. However, most studies have explored a single structure or local features. Few studies have focused on the analysis of the whole brain subregions, which might be restricted by the manual annotation method. However, it is extremely important to analyze the whole brain radiomic features using a machine learning method, because it can facilitate an objective and comprehensive evaluation of brain atrophy patterns, which may provide more effective and sensitive markers for the early diagnosis of AD.

Since different brain subregions can be affected by AD in a distinct manner, it is very essential to investigate the radiomic features of whole brain structures. In this study, by using a machine learning method, we first explored radiomic features of whole brain in different subregions between AD and HC and identified key subregions, which showed significant differences between the two groups. Second, we constructed classification models based on the radiomic features of selected subregions and different algorithms. Finally, by calculating the classification accuracy and evaluating the model performances, we identified the best model to predict AD. Based on the pathology of AD and previous studies, we hypothesized that this classification was driven by a distributed atrophy pattern of several subregions and mainly includes the hippocampus and other limbic systems, which might be affected early in the disease course. We expected that the model based on radiomic features of specific subregions can be applied as a valuable radiologic biomarker for the early diagnosis of AD.

## Materials and Methods

### Patient Information

In total, 160 right-handed subjects had participated in the study, i.e., 80 patients with AD and 80 healthy controls (HCs). This study was carried out in accordance with the recommendations of the Medical Research Ethics Committee of Aerospace Center Hospital. All subjects gave written informed consent in accordance with the Declaration of Helsinki. The protocol was approved by the Medical Research Ethics Committee of Aerospace Center Hospital. The AD subjects were recruited randomly from patients who had consulted the memory clinic at Aerospace Center Hospital for memory complaints. The HCs were recruited from the local community by recruitment advertisements. All the participants were required to complete the regular form, which includes age, gender, education, clinical history, family genetic history, previous examination results, and other clinical information.

All participants underwent a complete physical examination, neurological examination, and neuropsychological assessment. The neuropsychological examinations included the Mini-Mental State Examination (MMSE), the Clinical Dementia Rating (CDR) score, and other examinations. The patients with AD fulfilled the new research criteria for possible or probable AD (Dubois et al., 2007, 2010).

The HC fulfilled the following criteria: (a) no abnormal findings in routine brain Magnetic Resonance Imaging (MRI); (b) no findings of stroke, depression, or epilepsy, and other neurological or psychiatric disorders; (c) no visual loss or hearing loss and other neurological deficiencies; (d) no complaints about cognitive and memory; and (e) CDR score of 0.

The excluded criteria were as follows: participants with contraindications for MRI were excluded. For example, the subjects who have a cardiac defibrillator, a pacemaker, vascular clips, or a mechanical heart valve cannot take part in the examination; in addition, subjects with neurological or psychiatric diseases or with a history of cerebrovascular attacks or other degenerative disorders were excluded.

### Structural Magnetic Resonance Imaging Data Acquisition

Magnetic Resonance Imaging examinations were performed at the department of radiology using a 3.0T Siemens Skyra MR System (Siemens, Germany) with a 20-channel head coil. Sagittal T1-weighted structural images were acquired for each subject using a magnetization-prepared rapid gradient echo (MPRAGE) sequence. Three-dimensional (3D) MPRAGE sagittal images were obtained with following parameters: Time of Repetition (TR)/Time of Echo (TE)/Time of Inversion (TI)/Flip Angle (FA) = 1900 ms/2.2 ms/900 ms/9°, image matrix = 256 × 256, slice number = 176, and thickness = 1 mm. The obtained 3D images had a resolution of 1 mm × 1 mm × 1 mm.

### Segmentation and Evaluation of Subregions

The whole brain subregions of each patient were extracted automatically by the given deep learning model first. The model was trained by the United Imaging platform.^1^ The training process and the reference method of the model were similar to the previously published research (Desikan et al., 2006). The results of automatic segmentation included 22 temporal lobe structures, 20 frontal lobe structures, 12 parietal lobe structures, 8 occipital lobe structures, 8 cingulate gyrus structures, 2 insular structures, 12 subcortical gray matter structures, cerebral white matter structures, ventricles, the cerebellum, and other structures, with a total of 106 subregions. In particular, left and right structures were identified as different individuals. Once the automatic segmentation was done by the deep learning model, the result would be evaluated by two senior radiologists with more than 5 years of experience in radiologic diagnosis. In the segmentation results of 106 subregions of 160 patients in this study, two senior radiologists had no different opinions on the accuracy of the segmentation results.

### Radiomics Feature Extraction and Model Construction

**FIGURE 1:**
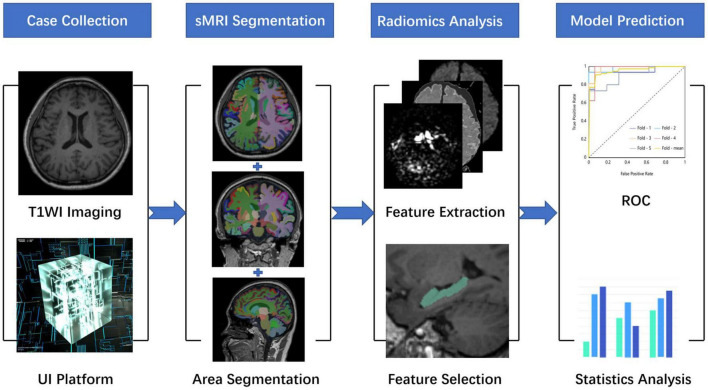
A research map. This study is mainly divided into four parts. The first part is data collection and preprocessing, the second part is whole-brain structure segmentation, the third part is radiomic analysis, and the fourth part is model construction and evaluation.

All preprocessing steps were performed using The United Imaging platform. Briefly, the radiomic features were extracted at first. Second, the features that were most consistent across different radiomics were selected to ensure robustness. Then, the dimension of extracted features was reduced using the Select K Best (K-Best) algorithm and traditional least absolute shrinkage and the selection operator (LASSO) algorithm, in which the two algorithms were used in series. Finally, the relevant parameters of these selected features would be used to build a machine learning model in order to successfully predict AD and HCs. These selected radiomic features of the most relevant subregions would be used in training sets and test sets in the form of 10-fold cross verification. The overall process is shown in Figure 1.

### Model Validation

**FIGURE 2:**
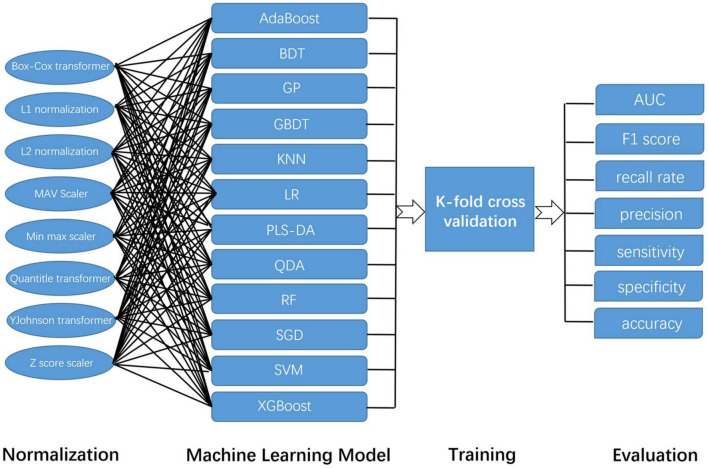
The construction and evaluation of the machine learning model in this study. Because this study adopts a variety of normalization methods and machine learning models, their combination is shown in this figure.

Before these data were used for model training, we used a variety of data normalization methods, such as Box-Cox transformer, L1 normalization, L2 normalization, max Absolute value scaler, min-max scaler, quantile transformer, YeoJohnson transformer, and Z score scaler. In this way, we tried to ensure the accuracy of the results. Then, we used a variety of common algorithms to predict AD and HCs, such as Adaptive Boosting (AdaBoost), Bagging Decision Tree (BDT), Gaussian Process (GP), Gradient Boosting Decision Tree (GBDT), K-Nearest Neighbor (KNN) algorithm, Logistic regression (LR), Partial Least Squares Discriminant Analysis (PLSDA), Quadratic Discriminant Analysis (QDA), RF, Stochastic Gradient Descent (SGD), SVM, and Extreme Gradient Boosting (XGBoost). The specific arrangement and combination ways are shown in Figure 2. The area under the curve (AUC) value, F1 score, recall rate, precision, sensitivity, specificity, and accuracy of each combination were evaluated separately.

### Statistical Analysis

Statistical analyses were performed using SPSS software 22.0 (IBM, Armonk, NY, United States). For numerical data in AD and HC groups, a Wilcoxon test was used to evaluate the differences between AD and HC groups. For categorical data, such as gender, a Fisher’s exact test was used to evaluate differences between AD and HC groups. Statistical significance was considered as *p* < 0.05.

## Results

### Basic Characteristics of the Patients

In total, 80 patients with AD and 80 HCs with high-resolution sMRI data were collected retrospectively, adjusted for age, sex, MMSE, and CDR, among others. The detail is shown in Table 1. There were no significant differences in age and sex between AD and HCs. There were significant differences in MMSE between the two groups.

**TABLE 1:** Clinical characteristics of AD patients and HC.

	AD(*N* = 80)	HC(*N* = 80)	*p*-value
Age, median(min–max)	65(46–88)	64.5(48–83)	*9.54 × 10^–1^
Sex, male/female	42/38	40/40	**9.87 × 10^–1^
MMSE, median(min-max)	15(0–25)	28(12–30)	*7.26 × 10^–5^
CDR	0.5–3	0	/

*Wilcoxon rank test;

**Fisher exact test. AD, Alzheimer’s disease; CDR, Clinical Dementia Rating; HC, healthy control; MMSE, Mini-Mental State Examination.

### Automatic Segmentation Results of Whole Brain Subregions

**FIGURE 3:**
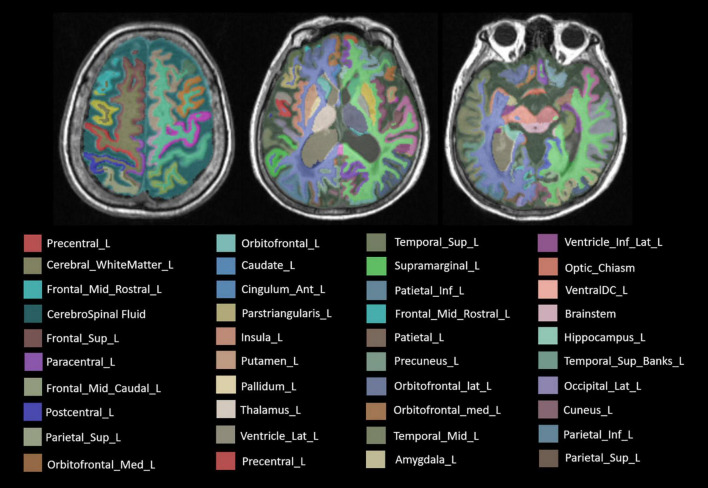
The label of main brain regions in structural MRI (sMRI) after automatic segmentation. Specially, the symmetrical structure is divided into left and right and has different labels. Only the structure on the left is marked above.

As the result, concrete 106 subregions included temporal lobe structures (the hippocampus, the para hippocampal gyrus, the amygdala, the entorhinal gyrus, the fusiform, the temporal pole, the superior temporal gyrus, the middle temporal gyrus, the inferior temporal gyrus, and the transverse temporal gyrus), frontal lobe structures (the precentral cortex, the superior frontal gyrus, the frontal middle rostral, the frontal middle caudal, the frontal pole, the lateral orbitofrontal lobe, the medial orbitofrontal lobe, the pars opercularis, the pars orbitalis, and the pars triangularis), the parietal lobe structures (the postcentral cortex, the paracentral cortex, the superior parietal lobule, vinferior parietal lobule, the precuneus, and the supramarginal gyrus), the occipital lobe structures (the cuneus gyrus, the lingual gyrus, the pericalcarine gyrus, and the lateral occipital gyrus), the cingulate gyrus (the anterior cingulate gyrus, the middle cingulate gyrus, the posterior cingulate gyrus, and the cingulate gyrus of isthmus), the insular lobe structures, the subcortical gray matter structures (the caudate, the putamen, the pallidum, the thalamus, the nucleus accumbens, and the claustrum), the cerebral white matter, ventricles (lateral ventricle, 3rd ventricle, 4th ventricle, and cerebrospinal fluid), the cerebellum (the cerebellum cortex and the cerebellum white matter), and other structures (the choroid plexus, the inferior horn of lateral ventricle, the brainstem, the optic chiasm, and the corpus callosum). The specific segmentation results of 106 brain areas are shown in Figure 3.

### Identification of Subregions Related to Alzheimer’s Disease

**FIGURE 4:**
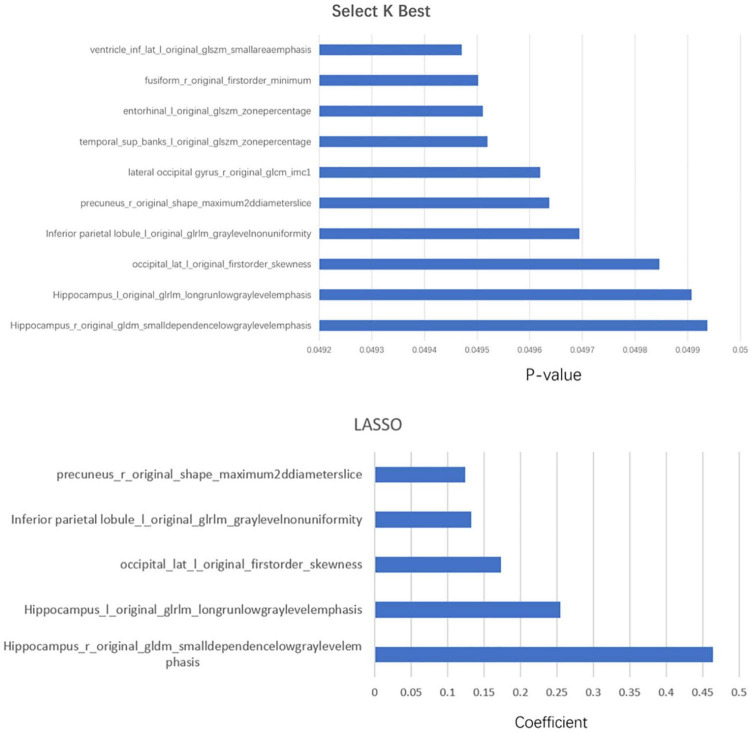
The combination of statistically significant brain regions and radiomic features in the two-step dimensionality reduction process. In the first step, Select K Best, more than 10 radiomic features, are screened out; only the top 10 are listed in this figure.

**FIGURE 5:**
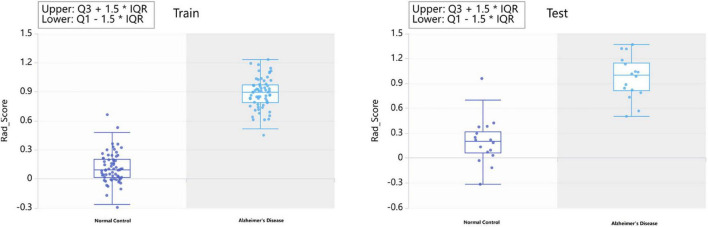
The rad score in radiomics feature selection.

Each subregion was extracted from 104 radiomic features and for a person, there would be, in total, 11,024 features with all 106 brain areas, which would be discussed in detail in the next section. We used two methods of dimensionality reduction in series, the Select K Best and LASSO. In the first step, we applied Select K Best to the 11,024 different features of subregions and screened the 3,660 specific features of subregions that may be related to AD (shown in Figure 4, only the top 10 features). Then, we applied LASSO to further screen the remained 3,660 specific radiomic features of subregions. After this, only 5 different radiomic features of 4 specific regions remained (shown in Figure 4). In the whole process of radiomic feature analysis, the rad scores of the training set and test set are shown in Figure 5.

As the above figures showed, there were several subregions related to AD. The most related subregions were the hippocampus, the inferior parietal lobule, the precuneus, and the lateral occipital gyrus.

### Identification of Important Radiomic Features

**FIGURE 6:**
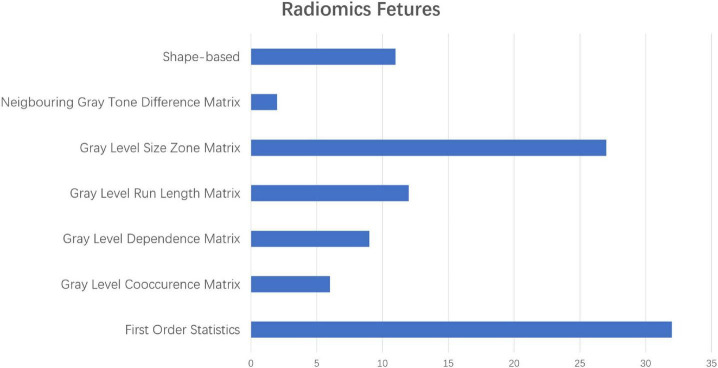
The different proportions of radiomic characteristics in the process of predicting ad with radiomic characteristics in the low-order dimension.

As mentioned above, some specific subregions played an important role in the prediction of AD. At the same time, different radiomic features of specific subregions also played different roles in the prediction of AD. In the dimension reduction process of Select K Best, we selected the relevant features of top 100 for further analysis. The low-order radiomic features we used include the following categories: first order statistics, shape-based features, gray level co-occurrence matrix (GLCM), gray level run length matrix (GLRLM), GLSZM, neighboring gray tone difference matrix (NGTDM), and GLDM. The result is shown in Figure 6. Furthermore, after LASSO screening analysis, we noticed that only 5 remained were the most important features in predicting AD and NCs, such as first order statistics, GLSZM (i.e., two different subtypes), shape, and GLDM, which are mentioned in Figure 5.

### The Evaluation and Comparison of Different Algorithms

We compared the performances of different algorithms for predicting AD. Table 2 summarizes the AUC, F1 score, recall rate, precision, sensitivity, specificity, and accuracy of the train set and test set using different algorithms. Each normalization method was listed at the model’s best performance. Especially, since we used the method of K-fold cross-validation, we got the above indicators for each training set and test set. Here, we use k-mean to reflect the average level of these models.

**TABLE 2:** Performance of different machine learning algorithms.

Model	Normalization	auc_train	auc_test	f1score_train	f1score_test	recall_train	recall_test	precision_train	precision_test	sensitivity_train	sensitivity_test	specificity_train	specificity_test	accuracy_train	accuracy_test
AdaBoost	Quantitle transformer	0.995	0.898	0.997	0.851	0.997	0.846	0.997	0.864	0.997	0.846	0.997	0.862	0.997	0.855
BDT	BoxCox transformer	0.995	0.951	0.995	0.909	0.990	0.910	1.000	0.913	0.990	0.910	1.000	0.912	0.995	0.911
GP	Min-max scaler	0.995	0.977	0.991	0.936	0.997	0.937	0.985	0.938	0.997	0.937	0.984	0.938	0.991	0.937
GBDT	BoxCox transformer	0.995	0.942	1.000	0.876	1.000	0.872	1.000	0.883	1.000	0.872	1.000	0.888	1.000	0.880
KNN	Z-score scaler	0.995	0.967	1.000	0.949	1.000	0.949	1.000	0.951	1.000	0.949	1.000	0.950	1.000	0.950
LR	BoxCox transformer	0.995	0.983	0.998	0.962	1.000	0.962	0.997	0.962	1.000	0.962	0.997	0.962	0.998	0.962
PLS-DA	Quantitle transformer	0.988	0.964	0.744	0.743	1.000	1.000	0.593	0.593	1.000	1.000	0.322	0.312	0.659	0.653
QDA	BoxCox transformer	0.974	0.969	0.934	0.935	0.924	0.923	0.945	0.949	0.924	0.923	0.947	0.950	0.936	0.937
RF	BoxCox transformer	0.989	0.966	0.958	0.936	0.949	0.936	0.968	0.938	0.949	0.936	0.969	0.938	0.959	0.937
SGD	Max-abs scaler	0.992	0.922	0.994	0.923	1.000	0.923	0.988	0.924	1.000	0.923	0.988	0.925	0.994	0.924
SVM	Z-score scaler	0.995	0.977	1.000	0.950	1.000	0.950	1.000	0.951	1.000	0.950	1.000	0.950	1.000	0.950
XGBoost	L1 normalization	0.995	0.957	1.000	0.903	1.000	0.898	1.000	0.911	1.000	0.898	1.000	0.912	1.000	0.906

*AdaBoost, Adaptive Boosting; BDT, Bagging Decision Tree; GP, Gaussian Process; GBDT, Gradient Boosting Decision Tree; KNN, K-Nearest Neighbor algorithm; LR, Logistic regression; PLSDA, Partial Least Squares Discriminant Analysis; QDA, Quadratic Discriminant Analysis; RF, random forest; SGD, Stochastic Gradient Descent; SVM, support vector machine; XGBoost, Extreme Gradient Boosting.*

**FIGURE 7:**
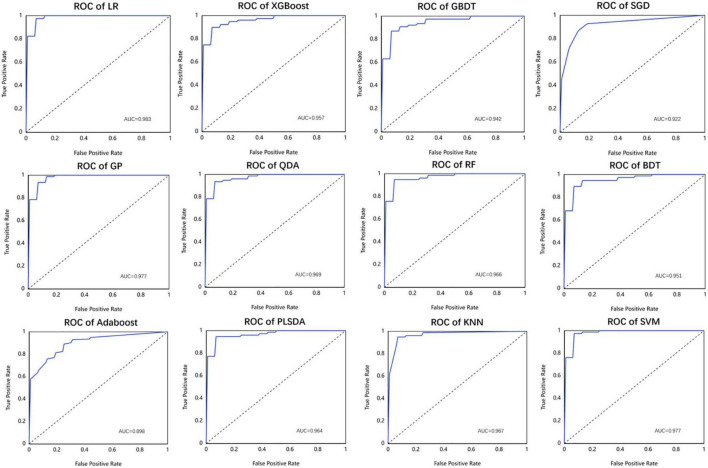
The performance of different machine learning models used in this study in the form of receiver operator curve (ROC).

As listed above, the best of all is an LR model with the Box-Cox transformer, which has an accuracy of 0.962 in the test set, followed by KNN of 0.950 and SVM of 0.950. The receiver operator curves (ROCs) of each model are listed in Figure 7.

## Discussion

For classifying AD and HC subjects, clinical evaluation (i.e., CDR and MMSE score) or imaging volume features were commonly used, which were not very accurate (Hu et al., 2016; Deters et al., 2017). Nomograms based on gene expression signatures, cerebral spinal fluid (CSF), and pathological features are not yet ready to be used in daily practice. Radiomic features extracted from MR scans provide a noninvasive means to predict AD (Wen et al., 2020; Yun et al., 2020; Feng et al., 2021).

In this study, we segmented the whole brain subregions of the enrolled cases and extracted the radiomic features of each segmented subregion. Then, we comprehensively analyzed all the radiomic features of the whole brain subregions and identified the subregions and radiomic features most related to AD. Using the relevant areas and radiomic parameters obtained, we built a variety of machine learning models, such as LR, SVM, and RF. Then, we evaluated the diagnostic efficiency of each model. Finally, we found the best model for predicting AD. In this study, the subregions most relevant to AD included the hippocampus, the inferior parietal lobe, the precuneus, and the lateral occipital gyrus. The radiomic features extracted from these subregions had the greatest differences between subjects with AD and HCs.

Among the four most relevant subregions found in our study, the relationship between the hippocampus and AD had been confirmed by many studies. The reduction of hippocampal volume had been well studied in individuals with AD (Stricker et al., 2012). Hippocampal atrophy was one of the core markers of AD in the revised national Alzheimer’s association diagnostic criteria (Albert et al., 2011; Catani et al., 2013). In addition to volume reduction, abnormal metabolic levels, interruption of brain activity, and microstructure characteristics in the hippocampus had also been well reported (Catani et al., 2013). The discriminative ability of radiomic features from hippocampal regions, found by our analysis, was consistent with recent studies (Sørensen et al., 2016, 2017). For example, the hippocampal texture was shown to be a strong biomarker for differentiating HCs from patients with AD or mild cognitive impairment (MCI) (Feng et al., 2018). Pathologically, a previous study has confirmed that hippocampal shape alterations were associated with regional Aβ load in normal elderly individuals (Schroeder et al., 2017). We could speculate that the radiomic feature changes of the hippocampus might result from AD pathological changes, such as Aβ deposition, which might be taken as a potential biomarker to differentiate between patients with AD and HCs.

The inferior parietal lobule had begun to show promise as an important locus in AD in recent years (Greene et al., 2010). For example, several studies had shown AD-related alterations of the inferior parietal lobule, such as gray matter atrophy (Desikan et al., 2009; Jacobs et al., 2011), metabolic dysfunction (Walhovd et al., 2010), disrupted spontaneous brain functional activity and connectivity (Wang et al., 2015), and pathological changes (Nelson et al., 2009). These findings had important implications for the underlying neurobiology of AD. Compared to previous studies, our analysis investigated the link between AD and radiomic features of inferior parietal lobule regions, which added a piece of new evidence for the mechanism of AD. In addition, our study might contribute to the early detection of AD to some extent.

As for the precuneus, its atrophy played a special role in early-onset AD (Karas et al., 2007). Many neuroimaging studies had demonstrated the structural and functional abnormalities of precuneus regions in AD, such as cortical thinning (Dickerson and Sperling, 2009), amyloid deposition (Buckner et al., 2009), decreased intrinsic brain activity (He et al., 2007; Wang et al., 2011), and disrupted functional connectivity of the region (Greicius et al., 2004). A previous fMRI study demonstrated reduced precuneus deactivation during object naming in patients with mild cognitive impairment, AD, and frontotemporal lobar degeneration (Frings et al., 2010). At the molecular level, precuneus amyloid burden was also associated with reduced cholinergic activity in AD (Ikonomovic et al., 2011), which might contribute to the cognitive decline. Our result on the link between AD and radiomic features in precuneus regions was consistent with the previous study, indicating the crucial role of precuneus in the early diagnosis of AD.

Finally, our study found that there is a specific relationship between the lateral occipital gyrus and AD. The machine learning model, which contained the lateral occipital gyrus, had higher diagnostic efficiency than the one without the lateral occipital gyrus constructed by radiomic features. The occipital gyrus is located in the primary visual cortex and plays a critical role in visual cognition. By using the fMRI method (Sala-Llonch et al., 2015), it was reported that the occipital gyrus presented higher activity during the task of visuo-perceptual working memory. Using diffusion tensor imaging (DTI) and tractography, a previous study demonstrated that the structural disconnection in the ventral occipital temporal cortex contributed to the deficit in facial recognition (Thomas et al., 2009). Visual cognition deficits were consistently reported to accompany the development of AD (Cronin-Golomb, 1995; Bokde et al., 2006). In our study, the potential relationship between the lateral occipital gyrus and AD put forward a possible new direction for the study of AD. The model constructed by integrating the most relevant structural areas provides a new idea for the prediction of AD.

However, there are still several issues that need further consideration in our study. First, in the current study, we mainly focused on structural analysis. Further studies that simultaneously combine the sMRI and other data, such as fMRI and clinical laboratory examination, might obtain a powerful and high-quality biomarker for clinical application. We plan to analyze the relationship between the radiomic features and the cognitive performances in the future to achieve early diagnosis and monitor the progress of the disease. Second, recent studies had paid more attention to individuals at high risk for AD, such as amnestic mild cognitive impairments, and ApoE-4 allele carriers. Exploring these populations would provide valuable biomarkers for the early diagnosis of AD. Finally, a longitudinal study with a large multicenter sample size is needed to confirm the stability and reliability of the model.

## Conclusion

In conclusion, our study specifically focused on the potential relationship between AD and the whole brain subregions based on sMRI. The machine learning model constructed with the radiomic features of the hippocampus, the inferior parietal lobe, the precuneus, and the lateral occipital gyrus could be used as a potential sMRI marker for predicting AD and had outstanding performance.

## Data Availability Statement

The original contributions presented in the study are included in the article/supplementary material, further inquiries can be directed to the corresponding author.

## Ethics Statement

The studies involving human participants were reviewed and approved by Ethics Review Committee of Aerospace Center Hospital. Written informed consent for participation was not required for this study in accordance with the national legislation and the institutional requirements.

## Author Contributions

SL: data acquisition, analysis, and interpretation for the research. CJ and WZ: image quality evaluation and evaluation of automatic segmentation results of whole brain areas. JC: extraction of radiomic features, construction of machine learning model, and parameter adjustment. ZW: drafting the work, providing final approval of the version to be published, and providing agreement to be accountable for all aspects of the work. All authors contributed to the article and approved the submitted version.

## Conflict of Interest

The authors declare that the research was conducted in the absence of any commercial or financial relationships that could be construed as a potential conflict of interest.

## Publisher’s Note

All claims expressed in this article are solely those of the authors and do not necessarily represent those of their affiliated organizations, or those of the publisher, the editors and the reviewers. Any product that may be evaluated in this article, or claim that may be made by its manufacturer, is not guaranteed or endorsed by the publisher.

## Footnotes

1
http://urp.united-imaging.com:8080/
